# Intra-marrow penetrations and root coverage outcomes: a systematic review

**DOI:** 10.1186/s12903-023-02964-6

**Published:** 2023-05-03

**Authors:** Vrisiis Kofina, Ying S. Wang, Alissa Fial, Dimitris N. Tatakis

**Affiliations:** 1 Department of Surgical Sciences, School of Dentistry, Marquette University, P.O. Box 1881, Milwaukee, WI 53201-1881 USA; 2 Division of Periodontology, College of Dentistry, The Ohio State University, 4121 Postle Hall, 305 W. 12th Ave, Columbus, OH 43210 USA; 3 Raynor Memorial Libraries, Marquette University, P. O. Box 3141, Milwaukee, WI 53201-3141 USA; 4 Department of Periodontics, School of Dentistry, Texas A&M University, 3302 Gaston Avenue, Dallas, TX 75246 USA

**Keywords:** Systematic review, Gingival recession, Bone marrow/*surgery, Osteotomy, Surgical flaps

## Abstract

**Background:**

Intra-marrow penetrations (IMPs) have been performed during guided tissue regeneration (GTR) procedures with reported clinical benefits. The aim of this systematic review was to investigate the use and effect of IMPs during root coverage procedures.

**Method:**

A broad search for human and animal studies was performed on PubMed, Cochrane Database of Systematic Reviews and Cochrane Central Registry of Controlled Trials and Web of Science, following a registered review protocol (PROSPERO). All prospective study designs, case series and case reports on gingival recession treatment (follow-up ≥ 6 months) that employed IMPs were included. Root coverage, complete root coverage prevalence, and adverse effects were recorded, and risk of bias was assessed.

**Results:**

Of 16,181 screened titles, 5 articles (all of them human studies) met inclusion criteria. All studies (including two randomized clinical trials) treated Miller class I and II recession defects, using coronally advanced flap with IMPs alone or in conjunction with GTR protocols. Therefore, all treated defects received IMPs and no studies compared protocols with and without IMPs. Outcomes were indirectly compared with existing root coverage literature. Mean root coverage was 2.7 mm and 68.5% at 6.8 months (median: 6 months, range 6–15 months) for sites treated with IMPs.

**Conclusion:**

IMPs are rarely used during root coverage procedures, have not been associated with intra-surgical or wound healing adverse effects and have not been investigated as independent factor. Future clinical studies are needed to directly compare treatment protocols with and without IMPs and investigate the potential benefits of IMPs for root coverage.

**Supplementary Information:**

The online version contains supplementary material available at 10.1186/s12903-023-02964-6.

## Introduction

Gingival recession defects, defined as the apical migration of the gingival/soft tissue margin resulting in exposure of the root surface to the oral environment, represent common mucogingival deformities that are highly prevalent in the population [1, 2]. Untreated gingival recession defects have a high likelihood of progressing over time [3]. Even though recession etiology is unclear, a thin periodontal phenotype, lack of attached gingiva, tooth malposition, physical trauma, orthodontic treatment, high frenal attachment, gingival inflammation, and periodontal disease are predisposing factors [4]. The recession-accompanying exposed root surfaces are often associated with dentinal hypersensitivity, esthetic concerns, and carious or non-carious cervical lesions. These conditions may prompt patients to seek treatment for root coverage [5]. Root coverage surgical techniques, which vary in flap design and their use of autogenous and non-autogenous grafts and biologic or synthetic materials, have been extensively investigated [6–8]. Although all procedures provide root coverage to a variable degree [7, 8], achievement of complete root coverage may depend on several factors, be they local, systemic, or technical, that modulate outcomes.

The effects of systemic (e.g., smoking [7, 9, 10]), local (e.g., carious and non-carious cervical lesions [11–13], and technical (e.g., suturing protocols [14]) factors on root coverage outcomes have been the focus of several studies. The most thoroughly investigated factors are related to soft tissue aspects, such as flap design [15], flap tension [16], and flap positioning [17]. In contrast, the hard tissue aspects of root coverage procedures have received comparatively limited attention and have been typically confined to studies on root surface modifications. More specifically, the role of mechanical root preparation [18, 19], deliberate reduction of root prominence [20], restoration of carious [11] or non-carious cervical lesions [12, 21], and the chemical modification of root surfaces (“root conditioning”) have also been investigated [3, 22, 23].

Despite the documented significance of alveolar bone anatomy for gingival recession, investigations on the possible effects of bone parameters are scarce. Buccal bone dehiscence depth is related to recession depth [24, 25] and has been among the investigated parameters during root coverage [26]. Similarly, buccal bone thickness has been associated with gingival recession [25]. Interproximal bone levels influence recession depth and progression [27] and have been used to classify recession defects and establish their prognosis [28, 29]. In contrast, there is little, if any, information on the potential impact of intentional bone modifications on root coverage outcomes.

One such common modification is intra-marrow penetrations (IMPs or alveolar decortications). The concept behind IMPs is that drilling through the cortical bone exposes bone marrow spaces, induces bleeding, and stimulates healing [30]. The potential clinical benefits of IMPs stem from the resulting natural and easier transport and access of undifferentiated mesenchymal cells and growth/differentiation factors to the treatment area; a growing body of research is focused on such approaches for the treatment of orofacial conditions such as alveolar bone regeneration and temporomandibular joint osteoarthritis [31]. The positive effects of IMPs on periodontal and peri-implant regenerative procedures have been documented [32, 33], as has been the absence of associated adverse effects [34, 35].

However, the potential impact of IMPs on root coverage outcomes has not been investigated. Therefore, the aim of this systematic review is to evaluate the literature on IMPs and root coverage outcomes for treatment of Miller class I, II and III gingival recession defects.

## Materials and methods

### Protocol and registration

This systematic review followed the PRISMA guidelines (PRISMA[36]) and was registered in the PROSPERO database (registration number CRD42020196120 for human studies and CRD42020220159 for animal studies).

### PICO question (population, intervention, comparison, outcome)

P: Humans and animals with maxillary and/or mandibular teeth with Miller class I, II and III buccal gingival recessions.

I: Root coverage procedure for treatment of gingival recession.

C: Root coverage outcomes between sites with and without IMPs.

O: Primary outcome: % and mm of root coverage at sites treated with and without IMPs.

Secondary outcome: % of sites achieving complete root coverage when treated with and without IMPs.

### Eligibility criteria

- Human studies.
- Animal studies.
- Age > 18 years (humans).
- Prospective clinical studies, randomized and non-randomized controlled clinical trials, case series, case reports.
- Follow-up of at least 6 months.
- Articles written in the English language.

### Exclusion criteria

- Retrospective studies.

### Information sources

A systematic literature search was developed with the guidance of a health sciences librarian (AF). The following databases were searched: PubMed, Cochrane Database of Systematic Reviews and Cochrane Central Registry of Controlled Trials, and Web of Science. The initial search was developed in PubMed using a combination of database-controlled vocabulary, Medical Subject Terms (MeSH) and keywords. The search was then refined based upon pre-selected articles relevant to the topic and the search question. Once the final search strategy was developed in PubMed, keywords were utilized to fit the parameters of the other databases.

The search parameters included a focus on decortication and regeneration terminology associated with gingival recession or root coverage procedures, specifically excluding terms related to intrabony or infrabony defects. Examples of terms used include “alveolar decortication,” “guided tissue regeneration,” “intra-marrow penetration,” “wound healing,” and “gingival recession”. The search in all databases was performed on 03/02/2022 and the complete final search strategies can be found in Supplementary file 1. In addition to the electronic searches (databases), hand searching was performed by reviewing the reference lists of the selected articles.

### Screening process, data extraction and analysis

**Fig. 1:**
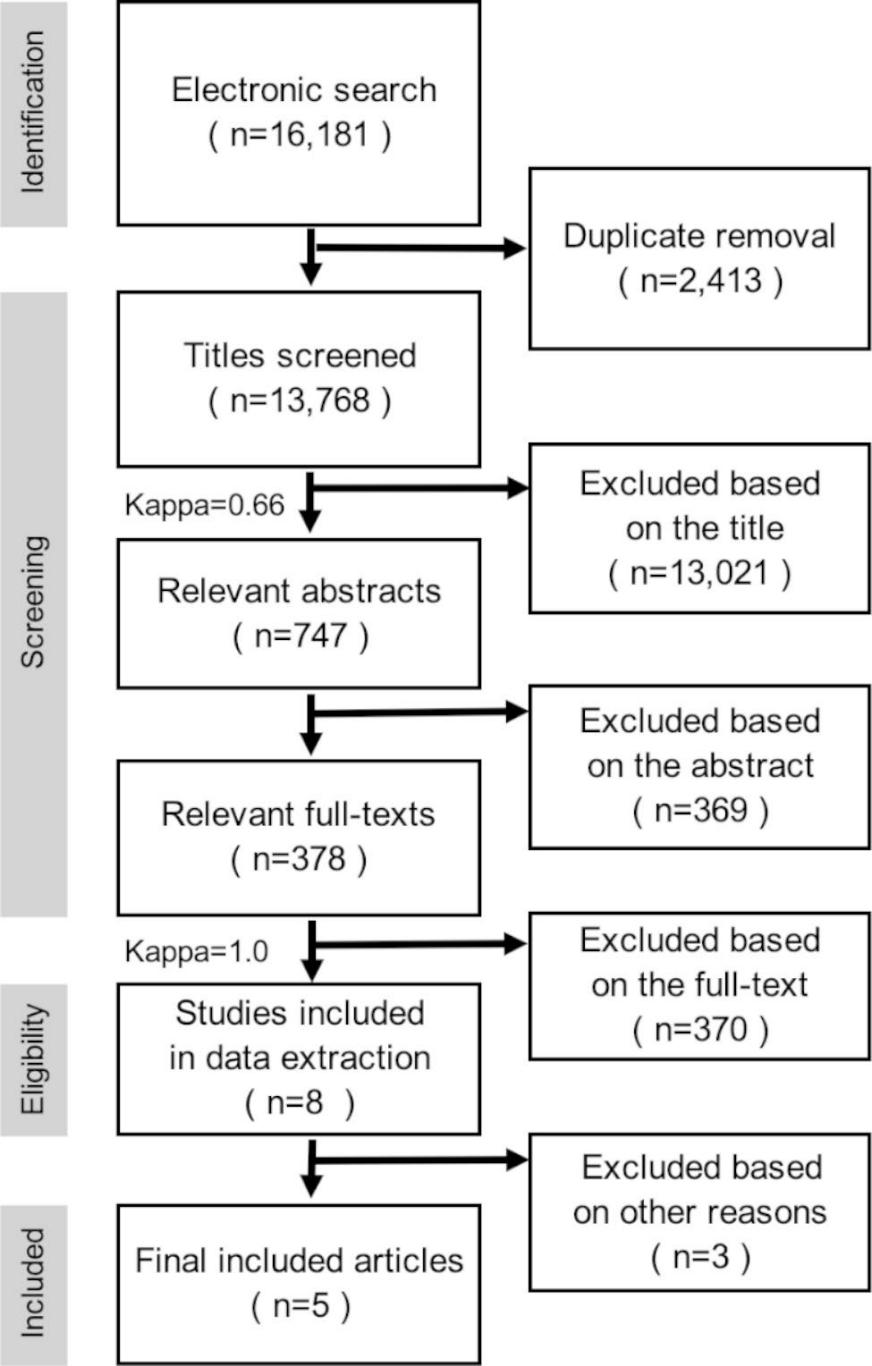
PRISMA flowchart illustrating the study selection process

Two reviewers (VK and YSW) independently screened titles, title-abstracts and full texts, and extracted data. In case of disagreements, consensus was reached by discussion with a third reviewer (DNT). The reasons for exclusion of studies were recorded. A flow diagram of the selection process is depicted in Fig. 1. Kappa coefficient was calculated to measure the level of agreement between the two reviewers (VK, YSW).

Data extraction included author, year, study design, Miller classification of treated defects, interventions (test and control), IMP protocol, sample size, participant smoking status, mm and % of root coverage, % of sites with complete root coverage, duration of study and adverse effects.

### Risk of bias assessment

Assessment for risk of bias for RCTs was performed using the revised Cochrane risk-of-bias tool for randomized trials (RoB 2 [37]).

The assessment was conducted at study level, independently by two reviewers (VK and YSW). A third reviewer (DNT) was consulted in case of disagreements.

## Results

### Literature search

The search strategies had a yield of 16,181 articles. After removing duplicates, the total number of articles was reduced to 13,768. Titles and abstracts were reviewed, and 13,021 articles were excluded. Out of the remaining 747, the full text of 378 articles was assessed and authors were contacted for missing data. Nine publications were identified that reported on IMP use in root coverage. One did not meet the inclusion criteria because it reported treatment of lingual recessions [38]. Another study fulfilled the inclusion criteria but did not clearly report root coverage outcomes and was therefore not included in the systematic review [39]. Two RCTs [40, 41] included the same patient population (9 out of 20 patients) with follow-up at different time points. Additionally, patients of a case series [42] were included in the patient population of a subsequently reported RCT [43]. In these two instances, the publications with the larger patient population were included in this systematic review. Finally, 5 studies fulfilled the eligibility criteria and were included in this systematic review. A flow diagram of the selection process is depicted in Fig. 1. Inter-examiner agreement was moderate at the title screening level (kappa = 0.66) and perfect at the full text-screening level (kappa = 1.0).

### Study characteristics

No animal studies met the inclusion criteria. Five clinical studies were included. The included study characteristics and results are presented in Tables 1 and 2. One study reported the results of a split-mouth randomized controlled clinical trial [40, 41], one study was a randomized controlled clinical trial [43], one was a case series [42, 44] and two were case reports [45, 46]. These five reports included a total of 75 treated recession defects in 52 patients (Tables 1 and 2). One study included both smokers and non-smokers [40, 41]. Among the other four studies, only one specified that the patient was a non-smoker [46]. All included recession defects were classified as Miller class I or II. The surgical protocol varied among studies. Recessions were treated with a coronally advanced flap alone [40, 41] or in conjunction with resorbable membrane [40, 41, 43], non-resorbable membrane [44], bone graft substitute and resorbable membrane [42, 43, 46] or autogenous bone-connective tissue graft and enamel matrix derivative [45]. Surgical outcome follow-up varied from 6 to 15 months (mean: 6.8 months; median: 6 months).

**Table 1:** Characteristics of included studies

Study	Study Design	Intervention			Miller Class	Smoking status	Duration
		Test	Control	IMP Protocol			
**Amarante**[40] **(2000)**	RCT	GTR + IMP (CAF + BM^†^)	CAF + IMP	“… small perforations were made by a round bur in the interdental bone areas to promote bleeding and stimulate bone marrow cell migration.“	I, II	8 smokers 12 non-smokers	6 months
	Split-mouth						
**Kimble**[43] **(2004)**	RCT	GTR + IMP (CAF + DFDBA + BM^‡^)	GTR + IMP (CAF + BM^‡^)	“… intra-bone marrow perforations were made on the mesial and distal portions of the root with a 1/2 round bur.“	I, II	Not Specified	6 months
	Parallel arm						
**Rocuzzo**[44] **(1996)**	Case series	GTR + IMP (CAF + NON-M^§^ + Miniscrews)	N/A	“Several holes were drilled into the cortical bone plate around the tooth to open the marrow space and to achieve a bleeding bone surface.“	I, II	Not Specified	9 months
**Nozawa**[45] ** (2002)**	Case report	CAF + CTB onlay graft + EMD + IMP	N/A	“The cortical bone of the buccal plate was removed in the interdental area using a 0.5-mm round bur to obtain sufficient blood supply.“	I	Not Specified	15 months
**Mukherji**[46] **(2016)**	Case report	GTR + IMP (CAF + CaSO4 graft + BM^†^)	N/A	“… made with a 1⁄2 round bur in the interproximal areas mesial and distal to the recipient teeth roots.“	I	Non-smoker	6 months

^†^Polylactic acid membrane ^‡^Collagen membrane ^§^Expanded polytetrafluorethylene membrane

Abbreviations: BM, bioabsorbable membrane; CAF, coronally advanced flap; CaSO4, calcium sulfate; CTB, connective tissue-bone; DFDBA, demineralized freeze-dried bone allograft; EMD, enamel matrix derivative; GTR, guided tissue regeneration; IMP, intra-marrow penetration; N/A, not applicable; Non-M: non-bioabsorbable membrane; RCT, randomized controlled trial

**Table 2:** Reported results of included studies

Study	Intervention		Sample size / Subjects		Sample size / Sites		Results		
	Test (T)	Control (C)	T	C	T	C	RC (mm)	RC (%)	CRC (% of sites)
**Amarante**[40] **(2000)**	GTR + IMP (CAF + BM^†^)	CAF + IMP	20	20	20	20	**T**: 2.3	56.1	25
							** C**: 2.5	69.4	50
**Kimble**[43] **(2004)**	GTR + IMP (CAF + DFDBA + BM^‡^)	GTR + IMP (CAF + BM^‡^)	10	8	10	8	**T**: 2.5 ± 0.5	74.3 ± 11.7	10
							** C**: 2.1 ± 0.9	68.4 ± 15.2	12.5
**Rocuzzo**[44] **(1996)**	GTR + IMP (CAF + NON-M^§^ + Miniscrews)	N/A	12	N/A	12	N/A	4.3	84	42
**Nozawa**[45] **(2002)**	CAF + CTB onlay graft + EMD + IMP	N/A	1	N/A	3	N/A	#8: 0	#8: 0	0
							#9: 1.5	#9: 60	
							#10: 3.0	#10: 75	
**Mukherji**[46] **(2016)**	GTR + IMP (CAF + CaSO4 graft + BM^†^)	N/A	1	N/A	2	N/A	N/A	#8: 100	50
								#9: 97	

^†^Polylactic acid membrane ^‡^Collagen membrane ^§^Expanded polytetrafluorethylene membrane

Abbreviations: BM, bioabsorbable membrane; CAF, coronally advanced flap; CaSO4, calcium sulfate; CRC, complete root coverage; CTB, connective tissue-bone; DFDBA, demineralized freeze-dried bone allograft; EMD, enamel matrix derivative; GTR, guided tissue regeneration; IMP, intra-marrow penetration; N/A, not applicable; Non-M: non-bioabsorbable membrane; RC, root coverage: mean ± *SD* (standard deviation); RCT, randomized controlled trial

### Outcome

All surgical protocols included IMPs for treatment of all recessions. Therefore, a comparison of root coverage outcomes between sites with and without IMPs was not possible. Root coverage amounted to a mean of 2.7 mm (68.5%). Overall mean frequency of complete root coverage was of 30.7%.

Adverse effects were reported only in one study [40] and were not related to the IMPs. In this split mouth study, one subject exhibited an inflammatory reaction 2 days postoperatively at the membrane site. Another subject had an orthodontic impression taken “during early healing”, affecting wound healing at both surgical sites.

Due to the lack of control treatment sites, meta-analysis was not performed.

### Risk of bias assessment

The risk of bias assessment of the two randomized controlled clinical trials is summarized in Table 3. The trial by Amarante et al. [40] was considered to have some concerns, whereas the trial by Kimble et al. [43] was considered to have a low risk of bias.

**Table 3:** Risk of bias assessment

	

## Discussion

This systematic review aimed to evaluate the effect of intra-marrow penetrations (IMPs) on root coverage outcomes. The results of the review, which is the first one to address the specific topic, indicate that although IMPs have been used in conjunction with root coverage procedures they have not been investigated as a potential outcome modifier. Therefore, a conclusion regarding the effect of IMPs on root coverage outcomes cannot be reached at this time; clinical trials aiming to investigate the potential effects of IMPs are necessary. The results of the review also indicate that use of IMPs in conjunction with root coverage surgical procedures does not result in any specific adverse effects, adding to the reported overall safety profile of this adjunctive procedure.

Although the first study utilizing IMPs in the course of a root coverage procedure was published in 1996, only a total of nine publications on this topic were identified in the current search and five articles met the eligibility criteria (Tables 1 and 2). Only Miller class I and II defects were treated in the included studies, and the overwhelming majority were treated with IMPs in the context of a guided tissue regeneration (GTR) procedure. IMPs have been employed in the course of GTR [33] and guided bone regeneration [34] approaches to treat intrabony and ridge deficiency defects, respectively. The available evidence suggests that adding IMPs to these regenerative surgical protocols is a safe and likely clinically beneficial modification [33, 34]. Treating gingival recession defects with various GTR approaches is a well-documented and critically investigated approach [7, 47–50]. In this context, the addition of IMPs to a surgical protocol using GTR to treat gingival recession defects is a rational and potentially outcome-enhancing approach. However, the lack of studies specifically assessing the contribution of IMPs to root coverage procedures, as documented in the present systematic review, precludes any conclusions regarding the usefulness of IMPs as an adjunct to surgical techniques used to treat gingival recession defects.

Study design and surgical protocol varied among the considered studies and only two studies (two clinical trials) included control treatments [40, 41, 43]. However, these studies, which aimed at comparing two different surgical protocols, did not evaluate the effect of IMPs; IMPs were performed on both test and control sites. Therefore, only indirect comparisons with published root coverage outcomes in the absence of IMPs are possible.

The 6-month mean root coverage for the included studies is comparable to published weighted mean coverage in systematic reviews on treatment of gingival recessions [7, 8, 51]. Percentage of root coverage, at 6 months postoperatively, in studies with IMPs was 69% [40] or 56–68% [40, 43] after coronally advanced flap (CAF) alone or with resorbable membrane, respectively. In other systematic reviews mean root coverage was 82.7% [51] and 55.9–95.4% for CAF alone [8], performed without IMPs. CAF plus a resorbable membrane led to 62.5–73.7% [8] mean root coverage. CAF plus non-resorbable membrane with miniscrews and IMPs led to 84% mean root coverage [52]. Similarly, the same approach without IMPs led to 80.5–82.4% in a recent systematic review [8].

50% [40] and 12.5–25% [40, 43] of sites achieved complete root coverage following CAF alone or with resorbable membrane in conjunction with IMPs, respectively. In comparison, 23.8–77.7% [51] or 7.7–81.8% of sites [8] exhibit complete root coverage without IMPs. The addition of a resorbable membrane led to 33.3–53.3% of sites having complete root coverage [8]. The use of CAF, non-resorbable membrane, miniscrews and IMPs resulted in 42% of sites having complete root coverage [44], whereas 28-41.6% of sites achieved complete root coverage after the use of CAF plus non-resorbable membrane, without IMPs [8]. Based on this limited data, it would appear that addition of IMPs does not critically alter CAF root coverage outcomes, whether positively or negatively. However, any definitive conclusions will require direct comparative trials.

The reported root coverage outcomes in the presence of IMPs may have been impacted by the small sample size and the inclusion of heavy smokers (> 20 cigarettes/day) in one clinical trial^35^. Smoking negatively affects wound healing by suppressing gingival flow and vascularity as well as delaying the proliferative phase of wound healing [53]. In a study on CAF for recession treatment, no sites achieved complete root coverage in smokers at 6 months, compared to 50% of sites in non-smokers [9]. Systematic reviews [7, 8, 54] have consistently reported that root coverage outcomes are poorer in smokers, especially those who smoke ≥ 10 cigarettes/day.

No adverse effects were reported specifically related to IMPs. The lack of IMP-related adverse effects in the present review is consistent with the reported lack of IMP-associated adverse events in the course of guided bone regeneration procedures [34, 35]. This suggests that properly performed IMPs, whose value as an adjunct for root coverage procedures remains to be determined, can be used without significant concerns. The potential positive effects of IMPs, which include histologically demonstrated early angiogenesis and osteogenesis during guided bone regeneration at edentulous sites [55–57] and whose benefits have been clinically documented during treatment of intrabony defects [33], may or may not be relevant for root coverage outcomes. Studies that include non-invasive (e.g., Cone-beam computed tomography) postoperative assessment of bone adjacent to IMP-treated recession defects would help determine the impact of IMPs in such clinical scenarios.

The main limitation of this systematic review is the small number of identified studies and their heterogeneity. IMPs were used only in a few studies and never as the investigated parameter. This is not surprising, as the focus of root coverage studies has been more on soft tissue parameters and systemic factors and less on hard tissue parameters [54, 58]. Another likely reason behind the lack of IMP-focused root coverage studies is the fact that GTR procedures [49, 51, 59], which target bone tissue and where IMPs may have an impact [33], have fallen out of favor as root coverage treatment modalities, given the common membrane-associated complications, such as membrane exposure [60], and the better short-[7, 61, 62] and long-term [7, 50] outcomes of other surgical techniques. Nevertheless, the present review, whose strengths include a broad and comprehensive search strategy and a focus on a previously ignored clinical topic, provides novel information which should help guide future clinical investigations.

## Conclusion

Use of IMPs during root coverage procedures is uncommon and is customarily associated with root coverage surgical techniques incorporating principles of GTR. The available limited evidence suggests that use of IMPs as an adjunct during root coverage procedures does not result in specific adverse effects. However, IMPs have not been investigated as a factor in root coverage studies and their potential impact on root coverage outcomes remains to be determined. Therefore, properly designed future studies are needed to assess the possible impact of IMPs on gingival recession treatment protocols.

## Electronic supplementary material

Below is the link to the electronic supplementary material.

Supplementary Material 1

## Data Availability

All relevant data from this study are included in the article and its Supplementary files. Further inquiries can be directed to the corresponding author.

## Abbreviations

GTR: Guided tissue regeneration
IMPs: Intra-marrow penetrations
RCT: Randomized controlled trial
CAF: Coronally advanced flap
