# Supplementary material for: Intra-marrow penetrations and root coverage outcomes: a systematic review
Source: BMC Oral Health. 2023 May 3;23:256. doi: 10.1186/s12903-023-02964-6 (PMC10157995; doi:10.1186/s12903-023-02964-6)
Supplement: Supplementary file 1 — Supplementary Material 1 [file 12903_2023_2964_MOESM1_ESM.docx]

**Supplementary file 1.** Search Strategy

**PubMed:**

(((“Alveolar decortication” OR Decortication* OR “intramarrow penetration” OR IMP OR “cortical penetration” OR “cortical perforation” OR "Periodontal Attachment Loss"[Mesh] OR “periodontal attachment loss” OR "Periodontal Attachment Loss/surgery"[Mesh] OR "Guided Tissue Regeneration"[Mesh] OR “guided tissue regeneration” OR "Guided Tissue Regeneration, Periodontal"[Mesh] OR “Guided Periodontal Tissue Regeneration” OR “Periodontal Guided Tissue Regeneration” OR "Bone Marrow/surgery"[Mesh] OR "Bone Regeneration"[Mesh] OR “bone regeneration” OR “bone regenerations”)) AND ("Wound Healing"[Mesh] OR “wound healing” OR “wound healings” OR “Soft tissue thickness” OR “gingival thickness” OR “mucosal thickness” OR “Attached gingiva” OR “attached mucosa” OR “keratinized tissue” OR “keratinized gingiva” OR “keratinized mucosa” OR “Mucogingival surgery” OR “root coverage” OR Esthetics OR "Esthetics, Dental"[Mesh] OR “Dental Esthetic” OR “Dental Esthetics” OR “Cosmetic Dentistry” OR “Cosmetic Surgery” OR “cosmetic surgeries” OR “soft tissue augmentation” OR “soft tissue transplantation” OR “soft tissue graft” OR “soft tissue grafts” OR “soft tissue grafting” OR “soft tissue autograft” OR “soft tissue correction” OR “free gingival graft” OR “subepithelial connective tissue graft” OR “connective tissue graft” OR "Connective Tissue/transplantation"[Mesh] OR “Connective tissue”[Mesh] OR “connective tissue” OR “connective tissues” OR “coronally advanced flap” OR “Coronally positioned flap” OR “laterally positioned flap” OR “double papilla flap” OR “Full-thickness flap” OR “full thickness flap” OR “Partial-thickness flap” OR “partial thickness flap” OR “Split-thickness flap” OR “split thickness flap” OR Emdogain OR "enamel matrix proteins"[Supplementary Concept] OR “enamel matrix proteins” OR “enamel matrix protein” OR “Enamel matrix derivative” OR “enamel matrix derivatives” OR biologics OR "Biological Products"[Mesh] OR “biological products” OR “biological product” OR “Natural Products” OR “natural product” OR Biopharmaceuticals OR “Biological Drugs” OR Biologicals OR “Biologic Medicines” OR “Biologic Pharmaceuticals” OR “Biologic Drugs” OR “Biological Medicines” OR "Platelet-Rich Fibrin"[Mesh] OR “Platelet-Rich Fibrin” OR “Platelet Rich Fibrin” OR “L-PRF” OR “Leukocyte- and Platelet-Rich Fibrin” OR “Leukocyte and Platelet Rich Fibrin” OR "Fibrin Tissue Adhesive"[Mesh] OR “fibrin tissue adhesive” OR “Fibrin Adhesive” OR “Fibrin Glue” OR “Fibrinogen Adhesive” OR “Fibrin Sealant System” OR “Autologous Fibrin Tissue Adhesive” OR “Fibrin Sealant” OR Crosseal OR Transglutine OR “Human Fibrin Sealant” OR Tisseel OR Tissel OR Tissucol OR Beriplast OR “Fibrin Seal” OR "Fibrin"[Mesh] OR Fibrin OR "Platelet-Rich Plasma"[Mesh] OR "Platelet-Rich Plasma" OR "Platelet Rich Plasma" OR "Membranes"[Mesh] OR Membrane OR Membranes OR “Membrane Tissue” OR “Membrane Tissues” OR “barrier membrane” OR “Barrier membranes” OR "Acellular Dermis"[Mesh] OR “Acellular dermis” OR “Acellular Dermal Tissue” OR “Acellular Dermal Tissues” OR “Acellular Dermal Graft Tissue” OR “Decellularized Dermal Scaffold” OR “Decellularized Dermal Scaffolds” OR “Acellular Dermal Matrix” OR “Acellular Dermal Matrices” OR “dermal matrix allograft” OR alloderm OR "Alloderm"[Supplementary Concept] OR “xenogeneic collagen matrix” OR “human fibroblast-derived dermal substitute” OR “human fibroblast derived dermal substitute” OR "Skin, Artificial"[Mesh] OR “artificial skin” OR “Artificial Skins” OR “Skin Substitutes” OR “Skin Substitute” OR dermagraft OR "Skin Transplantation"[Mesh] OR “skin transplantation” OR “Skin Grafting” OR “Skin Graftings” OR Dermatoplast* OR “Skin Transplantations” OR "Apligraf"[Supplementary Concept] OR apligraf OR “collagen matrix” OR "collagen matrix 10826"[Supplementary Concept] OR “CM-10826” OR "Collagen"[Mesh] OR Collagen OR Avicon OR Avitene OR “Collagen Felt” OR Zyderm OR Collastat OR Dermodress OR Pangen OR “alpha-Collagen” OR “alpha Collagen” OR “Collagen Fleece” OR Collagenfleece OR “extracellular membrane” OR "Gingival Recession"[Mesh] OR “gingival recession” OR “gingival recessions” OR "Gingival Recession/surgery"[Mesh] OR "Gingival Recession/therapy" OR “Gingival Atrophy” OR “Atrophy of Gingiva” OR "Tissue Conditioning, Dental"[Mesh] OR “dental tissue conditioning” OR “Tissue Conditioning (Dental)” OR "Surgical Flaps"[Mesh] OR “surgical flap” OR “surgical flaps” OR “Pedicled Flap” OR “Pedicled Flaps”)) NOT (Intrabony[ti] OR Infrabony[ti] OR “infra-bony” OR “intra-bony” OR “infra-bony”[ti] OR “intra-bony”[ti] OR Intrabony OR infrabony OR “Guided bone regeneration” OR “Alveolar ridge preservation” OR "Alveolar Ridge Augmentation"[Mesh] OR "Alveolar Ridge Augmentation" OR “Mandibular Ridge Augmentation” OR “Mandibular Ridge Augmentations” OR “Maxillary Ridge Augmentation” OR “Maxillary Ridge Augmentations” OR "Tooth Extraction"[Mesh] OR "Tooth Extraction" OR "Tooth Extractions" OR Trauma OR "Burns"[Mesh] OR Burn OR Burns OR “Intraosseous defect” OR “Intraosseous defects”)

**Filters:** English; Adult

**Cochrane Library (including Trials):**

(“Alveolar decortication” OR Decortication* OR “intramarrow penetration” OR IMP OR “cortical penetration” OR “cortical perforation” OR “periodontal attachment loss” OR “guided tissue regeneration” OR “Guided Periodontal Tissue Regeneration” OR “Periodontal Guided Tissue Regeneration” OR “bone regeneration*” OR “decortication of bone” OR “cortical perforation of bone” OR "Periodontal Attachment Loss surger*" OR "Bone Marrow surger*"):ti,ab,kw AND (“wound healing*” OR “Soft tissue thickness” OR “gingival thickness” OR “mucosal thickness” OR “Attached gingiva” OR “attached mucosa” OR “keratinized tissue” OR “keratinized gingiva” OR “keratinized mucosa” OR “Mucogingival surgery” OR “root coverage” OR Esthetics OR “Dental Esthetic*” OR “Cosmetic Dentistry” OR “Cosmetic Surger*” OR “soft tissue augmentation” OR “soft tissue transplantation” OR “soft tissue graft*” OR “soft tissue autograft” OR “soft tissue correction” OR “free gingival graft” OR “subepithelial connective tissue graft” OR “connective tissue graft” OR “connective tissue transplant*” OR “connective tissue*” OR “coronally advanced flap” OR “Coronally positioned flap” OR “laterally positioned flap” OR “double papilla flap” OR “Full-thickness flap” OR “full thickness flap” OR “Partial-thickness flap” OR “partial thickness flap” OR “Split-thickness flap” OR “split thickness flap” OR Emdogain OR “enamel matrix protein*” OR “Enamel matrix derivative*” OR biologics OR “biological product*” OR “natural product*” OR Biopharmaceuticals OR “Biological Drugs” OR Biologicals OR “Biologic Medicines” OR “Biologic Pharmaceuticals” OR “Biologic Drugs” OR “Biological Medicines” OR “Platelet-Rich Fibrin” OR “Platelet Rich Fibrin” OR “L-PRF” OR “Leukocyte and Platelet Rich Fibrin” OR “fibrin tissue adhesive” OR “Fibrin Adhesive” OR “Fibrin Glue” OR “Fibrinogen Adhesive” OR “Fibrin Sealant System” OR “Autologous Fibrin Tissue Adhesive” OR “Fibrin Sealant” OR Crosseal OR Transglutine OR “Human Fibrin Sealant” OR Tisseel OR Tissel OR Tissucol OR Beriplast OR “Fibrin Seal” OR Fibrin OR "Platelet-Rich Plasma" OR "Platelet Rich Plasma" OR Membrane* OR “Membrane Tissue*” OR “barrier membrane*” OR “Acellular dermis” OR “Acellular Dermal Tissue*” OR “Acellular Dermal Graft Tissue” OR “Decellularized Dermal Scaffold*” OR “Acellular Dermal Matrix” OR “Acellular Dermal Matrices” OR “dermal matrix allograft” OR alloderm OR “xenogeneic collagen matrix” OR “human fibroblast-derived dermal substitute” OR “human fibroblast derived dermal substitute” OR “artificial skin*” OR “Skin Substitute*” OR dermagraft OR “skin transplantation*” OR “Skin Graft*” OR Dermatoplast* OR apligraf OR “collagen matrix” OR "collagen matrix 10826" OR “CM-10826” OR Collagen OR Avicon OR Avitene OR “Collagen Felt” OR Zyderm OR Collastat OR Dermodress OR Pangen OR “alpha-Collagen” OR “alpha Collagen” OR “Collagen Fleece” OR Collagenfleece OR “extracellular membrane” OR “gingival recession*” OR "Gingival Recession surger*" OR “gingival recession therap*” OR “Gingival Atrophy” OR “Atrophy of Gingiva” OR “dental tissue conditioning” OR “surgical flap*” OR “Pedicled Flap*” OR “case report*” OR “case study” OR “case studies” OR “case histories” OR “case history”):ti,ab,kw NOT ((“infra-bony” OR “intra-bony” OR Intrabony OR infrabony OR “Guided bone regeneration” OR “Alveolar ridge preservation” OR "Alveolar Ridge Augmentation" OR “Mandibular Ridge Augmentation*” OR “Maxillary Ridge Augmentation*” OR "Tooth Extraction*" OR Trauma OR Burn OR Burns OR “Intraosseous defect*”)):ti,ab,kw" (Word variations have been searched)

**Web of Science:**

**TOPIC:** (“Alveolar decortication” OR Decortication* OR “intramarrow penetration” OR IMP OR “cortical penetration” OR “cortical perforation” OR “periodontal attachment loss” OR “guided tissue regeneration” OR “Guided Periodontal Tissue Regeneration” OR “Periodontal Guided Tissue Regeneration” OR “bone regeneration*” OR “decortication of bone” OR “cortical perforation of bone” OR "Periodontal Attachment Loss surger*" OR "Bone Marrow surger*") *AND* **TOPIC:** (“wound healing*” OR “Soft tissue thickness” OR “gingival thickness” OR “mucosal thickness” OR “Attached gingiva” OR “attached mucosa” OR “keratinized tissue” OR “keratinized gingiva” OR “keratinized mucosa” OR “Mucogingival surgery” OR “root coverage” OR Esthetics OR “Dental Esthetic*” OR “Cosmetic Dentistry” OR “Cosmetic Surger*” OR “soft tissue augmentation” OR “soft tissue transplantation” OR “soft tissue graft*” OR “soft tissue autograft” OR “soft tissue correction” OR “free gingival graft” OR “subepithelial connective tissue graft” OR “connective tissue graft” OR “connective tissue transplant*” OR “connective tissue*” OR “coronally advanced flap” OR “Coronally positioned flap” OR “laterally positioned flap” OR “double papilla flap” OR “Full-thickness flap” OR “full thickness flap” OR “Partial-thickness flap” OR “partial thickness flap” OR “Split-thickness flap” OR “split thickness flap” OR Emdogain OR “enamel matrix protein*” OR “Enamel matrix derivative*” OR biologics OR “biological product*” OR “natural product*” OR Biopharmaceuticals OR “Biological Drugs” OR Biologicals OR “Biologic Medicines” OR “Biologic Pharmaceuticals” OR “Biologic Drugs” OR “Biological Medicines” OR “Platelet-Rich Fibrin” OR “Platelet Rich Fibrin” OR “L-PRF” OR “Leukocyte- and Platelet-Rich Fibrin” OR “Leukocyte and Platelet Rich Fibrin” OR “fibrin tissue adhesive” OR “Fibrin Adhesive” OR “Fibrin Glue” OR “Fibrinogen Adhesive” OR “Fibrin Sealant System” OR “Autologous Fibrin Tissue Adhesive” OR “Fibrin Sealant” OR Crosseal OR Transglutine OR “Human Fibrin Sealant” OR Tisseel OR Tissel OR Tissucol OR Beriplast OR “Fibrin Seal” OR Fibrin OR "Platelet-Rich Plasma" OR "Platelet Rich Plasma" OR Membrane* OR “Membrane Tissue*” OR “barrier membrane*” OR “Acellular dermis” OR “Acellular Dermal Tissue*” OR “Acellular Dermal Graft Tissue” OR “Decellularized Dermal Scaffold*” OR “Acellular Dermal Matrix” OR “Acellular Dermal Matrices” OR “dermal matrix allograft” OR alloderm OR “xenogeneic collagen matrix” OR “human fibroblast-derived dermal substitute” OR “human fibroblast derived dermal substitute” OR “artificial skin*” OR “Skin Substitute*” OR dermagraft OR “skin transplantation*” OR “Skin Graft*” OR Dermatoplast* OR apligraf OR “collagen matrix” OR "collagen matrix 10826" OR “CM-10826” OR Collagen OR Avicon OR Avitene OR “Collagen Felt” OR Zyderm OR Collastat OR Dermodress OR Pangen OR “alpha-Collagen” OR “alpha Collagen” OR “Collagen Fleece” OR Collagenfleece OR “extracellular membrane” OR “gingival recession*” OR "Gingival Recession surger*" OR “gingival recession therap*” OR “Gingival Atrophy” OR “Atrophy of Gingiva” OR “dental tissue conditioning” OR “surgical flap*” OR “Pedicled Flap*” OR “case report*” OR “case study” OR “case studies” OR “case histories” OR “case history”) *NOT* **TITLE:** (Intrabony OR Infrabony OR “infra-bony” OR “intra-bony”) *NOT* **TOPIC:** (“infra-bony” OR “intra-bony” OR Intrabony OR infrabony OR “Guided bone regeneration” OR “Alveolar ridge preservation” OR "Alveolar Ridge Augmentation" OR “Mandibular Ridge Augmentation*” OR “Maxillary Ridge Augmentation*” OR "Tooth Extraction*" OR Trauma OR Burn OR Burns OR “Intraosseous defect*”)

**Refined by:** **LANGUAGES:** ( ENGLISH )

**Timespan:** All years. **Indexes:** SCI-EXPANDED, SSCI, A&HCI, ESCI.
